# Postoperative complications after surgery for lumbar spinal stenosis, assessment using two different data sources

**DOI:** 10.1007/s00701-024-06086-y

**Published:** 2024-04-23

**Authors:** Ole Kristian Alhaug, Filip C. Dolatowski, Simran Kaur, Greger Lønne

**Affiliations:** 1 The Research Center for Age-Related Functional Decline and Disease, Innlandet Hospital Trust, PO Box 68, N-2313 Ottestad, Norway; 2 Orthopaedic Department, Akershus University Hospital, PO Box 1000, N-1478 Loerenskog, Norway; 3 Orthopaedic Department, Oslo University Hospital, PO Box 4956, N-0424 Oslo, Norway; 4 Orthopedic Department, Martina Hansens Hospital, Dønskiveien 8, 1346 Gjettum, Norway; 5 Innlandet Hospital Trust, PO Box 104, N-2381 Brumunddal, Norway

**Keywords:** Lumbar spinal stenosis, Postoperative complications, Spine registry, Electronic patient record, Data accuracy

## Abstract

**Purpose:**

Lumbar spinal stenosis (LSS) is a prevalent disorder, and surgery for LSS is a common procedure. Postoperative complications occur after any surgery and impose costs for society and costs and additional morbidity for patients. Since complications are relatively rare, medical registries of large populations may provide valuable knowledge. However, recording of complications in registries can be incomplete.

To better estimate the true prevalence of complications after LSS surgery, we reviewed two different sources of data and recorded complications for a sample of Norwegian LSS patients.

**Methods:**

474 patients treated surgically for LSS during 2015 and 2016 at four hospitals reported to a national spine registry (NORspine).

Postoperative complications were recorded by patients in NORspine, and we cross-referenced complications documented in NORspine with the patients´ electronic patient records (EPR) to re-test the complication rates. We performed descriptive statistics of complication rates using the two different data sources above, and analyzed the association between postoperative complications and clinical outcome with logistic regression.

**Results:**

The mean (95%CI) patient age was 66.3 (65.3–67.2) years, and 254 (53.6%) were females. All patients were treated with decompression, and 51 (10.7%) received an additional fusion during the index surgery. Combining the two data sources, we found a total rate for postoperative complications of 22.4%, the NORspine registry reported a complication rate of 15.6%, and the EPR review resulted in a complication rate of 16.0%. However, the types of complications were inconsistent across the two data sources. According to NORspine, the frequency of reoperation within 90 days was 0.9% and according to EPR 3.4%. The rates of wound infection were for NORspine 3.1% and EPR review 2.1%.

There was no association between postoperative complication and patient reported outcome.

**Conclusion:**

Postoperative complications occurred in 22% of LSS patients. The frequency of different postoperative complications differed between the two data sources.

## Introduction

Lumbar spinal stenosis (LSS) is a prevalent disorder, and surgery for LSS is a common procedure estimated to increase with an ageing population [25, 29]. Frequencies of postoperative complications after surgery for LSS have been reported between 2,1 and 21,9% [5, 10, 26]. The large gap in frequencies can have several reasons; registration of postoperative complications is complex; the patients are often discharged from the treating centre when the postoperative complication occurs, and postoperative complications may go untreated or may be treated by other healthcare institutions than the treating center (i.e., primary doctor, emergency care unit), hence some postoperative complications may go unnoticed by the treating centre. Furthermore, there are no common standards reporting postoperative complications [18]. Prospective clinical cohort studies with closer follow-up may uncover more postoperative complications, but such studies include relatively few patients and may miss rare complications. Medical registries include large populations and are well situated to detect rare complications; however, registries often have a considerable loss to follow-up, and the results may be biased by inaccurate recording and underestimating perioperative complications have been reported [3, 16].

Postoperative complications occur after any surgery and impose societal and individual costs as well as additional morbidity. Accurate information about complications is essential to make informed choices about surgical treatment, and heterogeneity in definitions and methods for recording postoperative complications has been pointed at as a problem [15, 19, 22].

Even if postoperative complications usually can be treated and healed, some studies have shown inferior patient satisfaction among patients who suffered complications [4, 14, 21]. We aimed to estimate the risk of postoperative complications after surgery for LSS using two different data sources to better estimate the frequency, and to demonstrate the differences between the two methods for recording postoperative complications. Furthermore, we aimed to assess the association between postoperative complications and clinical outcomes.

## Methods

Retrospective study based on prospectively collected register and electronic patient record (EPR) data. We included patients treated for LSS at four selected hospitals during 2015 and 2016 who consented and reported on outcomes, satisfaction, and complications to the NORspine registry. NORspine is a mandatory national registry for degenerative spine surgery, covering all spine treatment centres in Norway [17]. Ideally, all patients receiving spine surgery in Norway are included in the registry; however, the capture rate is about 74% [17]. The exclusion criteria in the NORspine registry are patients unable to give informed consent, aged under 18 years, patients with severe psychiatric diagnoses or drug problems, as well as patients treated for spinal tumours, fractures, or infections.

The registry includes preoperative information regarding symptoms and disability using common patient-reported outcome measures (PROMs) and socioeconomics. The registry also includes surgeon-reported variables regarding diagnosis and surgical details (including perioperative complications and reoperations within 90 days), radiologic findings, and relevant comorbidities. Clinical outcome is recorded by the patients after three — and twelve months using common PROMS, a transitional scale (Global Perceived Effect (GPE) and patient satisfaction [17]. The Global Perceived Effect (GPE) is a seven-step scale on clinical change (“worse than ever”, “much worse”, “somewhat worse”, “unchanged”, “somewhat better”, “much improved”, and “ completely recovered”) [12]. Surgeons also reported intraoperative complications as well as any reoperations done within 90 days of the index surgery.

In NORspine, postoperative complications are recorded by the patients themselves at three months follow-up, and the follow-up rate in NORspine is 72% [17].

We also identified any postoperative complications by reviewing Electronic Patient Records (EPRs) at the four treating centres. The EPR reviews were done by two of the authors, and the interrater reliability was tested and found to be excellent [3]. In EPRs, complications are recorded by healthcare personnel; however, there are no standardized follow-up intervals.

The prevalence of postoperative complications was then estimated by combining the numbers derived from the two data sources (NORspine and EPR).

### Statistics

Baseline data were described using mean (95%CI) and numbers (proportions (%)). Clinical outcome was described using mean (95%CI) and numbers (proportions (%)), failure was defined by GPE (“worse” or “worse than ever” [2]. Postoperative complications were described using numbers (proportions (%)). The association between postoperative complications and patient satisfaction and clinical outcome was calculated with logistic regression adjusting for confounding factors (Table 4).

The calculations were done by SPSS version 26 (IBM Corp. released in 2017. IBM SPSS Statistics for Windows, version 26. Armonk, NY, USA).

This study was approved by the national ethical board (2017/2157) and the data protection offices at the involved hospitals. The study was conducted in accordance with the Helsinki Declaration, and we have presented the results in line with the STROBE guidelines [28].

## Results

We included 474 patients, and 327 (69.0%) reported to NORspine at three months follow-up. Table 1 displays baseline variables and surgical details of the study population at baseline and three months after LSS surgery. The mean (95%CI) age was 66.3 (65.3–67.2) years, and 254 (53.6%) were females.. The mean (95%CI) preoperative ODI was 40.9 (39.5–42.4). Fifty-one (10.7%) had a fusion procedure in addition to decompression.

**Table 1:** Patient characteristics and treatment for 474 Norwegian patients with surgically treated lumbar spinal stenosis vs 326 patients that completed 3 months follow up

	All (*n* = 474) Mean (95%CI), or n (%)	Completed 3 mths FU (*n* = 327) Mean (95%CI), or n (%)
Age	66.3 (65.3 – 67.2)	67.4 (66.3 – 68.6)
Female gender	254 (53.6%)	182 (55.7%)
Civil status, single	115 (24.3%)	79 (24.2%)
Norwegian as 1st language	454 (95.8%	312 ((95.4%)
ASA (grade 3 to 5)*	121(25.5%)	87 (26.7%)
Body Mass Index	28.3 (27.9 – 28.7)	28.2 (27.7 – 28.7)
Smoking	111 (23.7%)	68 (21.0%)
University or college education	113 (23.8%)	76 (23.2%)
Receives Disability benefit	63 (13.3%)	42 (12.8%)
Leg pain > 12 months before surgery	276 (58.2%)	181 (55.4%)
Preoperative ODI**	40.9 (39.5 – 42.4)	40.6 (38.8 – 42.3)
Preoperative NRS leg pain***	6.96 (6.76 – 7–16)	7.06 (6.83 – 7.28)
Preoperative NRS back pain***	6.80 (6.60 – 6.99)	6.77 (6.53 – 7.01)
Preoperative EQ-5D****	0.332 (0.302 – 0.362)	0.340 (0.304 – 0.376)
Previous spinal surgery (same level)	131 (27.8%)	82 (25.4%)
Additional fusion (any type)	51 (10.7%)	29 (8.9%)
More than one level operated	145 (30.6%)	102 (31.3%)

^*^ American Society of Anesthesiologists classification (1–5), increasing for worse health

^**^Oswestry Disability Index (0 – 100), increasing for increasing disability

^***^Numeric Rating Scale (0 – 10), increasing for increasing pain

^****^EuroQol´s quality of life (-0.60 – 1.00), increasing for better quality of life

Clinical outcomes are displayed in Table 2; the mean (95%CI) ODI score after three months was 24.8 (23.0–26.7). Fifty-four patients (16.6%) graded themselves as treatment failures by GPE (no improvement or worse), and 18 (4.8%) were dissatisfied with the treatment.

**Table 2:** Clinical outcome for 326 patients treated surgically for LSS at 4 hospitals in Norway in 2015 + 2016 and completed 3 months follow-up. Mean (95%CI) or number (proportion)

ODI final score *	24.8 (23.0 – 26.7)
NRS leg pain **	3.57 (3.26 – 3.88)
NRS back pain **	4.04 (3.75 – 4.33)
EQ5D ***	0.615 (0.581 – 0.650)
Failure ****	54 (16.6%)
Dissatisfied *****	18 (4.8%)

^*^ Oswestry Disability Index (0–100), increasing disability with increasing number

^**^ Numeric Rating Scale (0–10), increasing pain with increasing number

^***^EuroQol 5 Dimentions (-0.6- – 1.00), increasing quality of living with increasing number

^****^Defined as Global Perceived Effect (GPE) 4 + 5 + 6 + 7 (unchanged or any degree of worsening) at 3 months

^*****^Defined as some — or very dissatisfied at 3 months

The frequencies of postoperative complications are displayed in Table 3. According to patient-reported NORspine data, 51/326 (15.6%) recorded any postoperative complication, and the corresponding frequency according to EPR was 76/474 (16.0%). However, the two data sources did not match completely, and by combining NORspine and EPR data, we found a total postoperative complication frequency of 106/474 (22.4%).

**Table 3:** Postoperative complications in 474 patients surgically treated for LSS in 2015 and 2016 at 4 hospitals in Norway, EPR-registered vs patient-registered (NORspine)

Postoperative complications	EPR *n* = 474	NORspine *n* = 326*	Combined** (*n* = 474)
Hemorrage	3 (0.6%)	0 (0.0%)	3 (0.6%)
Deep venous Thrombosis	1 (0.2%)	1 (0.3%)	1 (0.2%)
Wound infection, superficious	6 (1.3%)	9 (2.8%)	13 (2.7%)
Wound infection, deep	4 (0.8%)	1 (0.3%)	4 (0.8%)
Pulmonary embolism	2 (0.4%)	2 (0.6%)	3 (0.6%)
Pneumonia	8 (1.7%)	6 (1.8%)	12 (2.5%)
Micturition problems	17 (3.6%)	21 (6.4%)	32 (9.8%)
Urinary tract infection	13(2.7%)	15 (4.6%)	22 (4.6%)
Epidural hematoma***	16 (3.4%)		
Pain / neurologic deficit***	21 (4.4%)		
Total	**76 (16.0%)**	**51 (15.6%)**	**106 (22.4%)**
Readmission ***	**21 (4.4%)**		
Re-operation within 90 days	**16 (3.4%)**	**3 (0.9%)**	**18 (3.8%)**

^*^326 of 474 answered 3 months follow-up

^**^In combined, every complication is counted only once, not only adding EPR and NORspine. Percentages are based on 474 patients

^***^ Epidural hematoma, pain, neurologic deficit and readmission are only registrated in EPR

Seventeen (3.5%) of the patients had a postoperative infection; however, patients classified infections differently (NORspine) than healthcare personnel (EPR). According to NORspine, 3 (0.9%) of patients had a reoperation within 90 days of the index surgery; the corresponding number was 16 (3.4%), according to the EPR review.

Twenty-one (6.4%) patients reported postoperative micturition problems to NORspine vs. 17 (3.6%) identified by the EPR review. Similarly, patients more often reported urinary tract infection (UTI) to NORspine 15 (4.6%) compared to 13 (2.7%) documented UTI by health care personnel in corresponding EPRs.

Patients were not asked to report postoperative epidural hematoma, pain/neurologic deficit, or readmission to NORspine; however, EPRs documented epidural hematoma in 16 (3.4%) patients, postoperative pain or neurologic deficit in 21 (4.4%) patients, and readmission for 21 (4.4%) patients.

We found no significant difference in ODI score (95%C I) at twelve months follow-up between patients suffering from postoperative complications or not; mean ODI (95%CI) was(28.2 (23.7–32.6) vs 24.4 (22.1–26.7); *p* = 0.131 (MD (95%CI) = 3.8 (-1.1 – 8.7)), and we found no association between postoperative complication and patient-reported clinical outcome three months after surgery (Table 4).

**Table 4:** Association between postoperative complications (combined) and failure (Global Perceived Effect = “worse” and “worse than ever”) and dissatisfaction at 3 months after surgery for lumbar spinal stenosis adjusted for potential confounders

	failure		dissatisfied	
	OR (95%CI)	*p*-value	OR (95%CI)	*p*-value
Postoperative complication	**0.60 (0.26–1.39)**	**0.235**	**1.24 (0.34–4.55)**	**0.747**
Age	0.52 (0.95–1.03)	0.517	1.02 (0.96–1.09)	0.553
Gender (man)	1.62 (0.82–3.19)	0.163	1.52 (0.47–4.90)	0.484
Smoking	1.02 (0.45–2.32)	0.956	1.27 (0.28–5.76)	0.757
BMI	1.03 (0.95–1.11)	0.487	0.97 (0.83–1.13)	0.685
ASA classification	1.14 (0.58–2.25)	0.702	0.63 (0.19–2.05)	0.445
ODI preopr	1.01 (0.99–1.04)	0.282	0.98 (0.94–1.03)	0.479
NRS backpain preopr	1.01 (0.79–1.28)	0.957	1.44 (0.88–2.36)	0.145
NRS leg pain preopr	0.89 (0.70–1.13)	0.341	0.66 (0.42–1.04)	0.071
Symptom duration over 12m	1.09 (0.51–2.34)	0.823	0.78 (0.22–2.86)	0.713
Additional fusion	0.54 (0.15–1.99)	0.357	2.50 (0.56–11.20)	0.230
Previous spine surgery	2.47 (1.21–5.04)	0.013	5.17 (1.54–17-33)	0.008
Number of levels operated	0.64 (0.34–1.19)	0.161	0.92 (0.30–2.79)	0.882

## Discussion

This retrospective study on prospectively collected registry data and EPRs documented postoperative complications in 51 (15.6%) and 76(16.0%) LSS patients, respectively. When the two data sources were combined, we identified that 106 (22.4%) of the LSS patients had suffered any postoperative complication. Patients more often reported micturition problems (including UTI) to NORspine compared to healthcare personnel-recorded micturition problems documented in the corresponding EPRs. At the same time, re-operations within 90 days were more often recorded in the EPR than in the registry. Infections were recorded somewhat differently by patients (NORspine) and healthcare personnel.

The rates of postoperative complications after LSS surgery have been reported between 2–20% [5, 10, 26]. Studies use different definitions of postoperative complications and the methods of recording complications are usually based on EPR review, register data or patient-completed forms. This may explain differences in reported frequencies of postoperative complications. Registries more reliably identify rare complications because of the large sample size; however, register data may suffer from attrition bias and inaccurate recording [3, 16]. Awareness of attrition bias and underreporting is essential when interpreting complications recorded in medical registries.

In our study, patients reported (NORspine) postoperative micturition problems more often than the corresponding EPR supplied by health care personnel. Other studies have found rates of micturition problems after LSS surgery of 0.4- 17% [5, 8, 26, 30]. The discrepancy between the two data sources may be explained by the theory that most micturition problems and UTIs do not need hospitalization and are, therefore, not registered in hospital records (EPRs). Hence, we consider patients (NORspine) the most reliable source for this variable; however, both data sources underestimate the prevalence according to the combined estimate.

Postoperative infections occurred in 17 (3.5%) patients; previously published data estimate the frequency of infection between 0.1% and 4.2% [5, 8, 26]. We also found discrepancies regarding the frequencies of postoperative infections after LSS surgery. Patients (NORspine) more often reported superficial infections than the corresponding number documented in EPRs. This discrepancy may be caused by some superficial infections being treated ambulatory by GPs. On the contrary, EPRs recorded more deep infections than the patients (NORspine). This discrepancy may be caused by patients not being able to discern between different grades of infection.

Our EPR review discovered more re-operations than caught by the NORspine registry, and the magnitude of the difference was somewhat surprising. The frequency of re-operations based on EPR aligns with previously published studies that report frequencies of revision surgery between 3.3% and 8.3% [5, 26, 32]. The frequency of revision surgery reported by NORspine (0.9%) is far below the EPR frequency and other reports [5, 26, 32]. In NORspine, surgeons report re-operations done within 90 days of the index surgery, and a previous NORspine study has questioned the accuracy of certain surgeon-reported variables [3]. We believe that EPRs are the more reliable source regarding re-operations and that NORspine (patient-reported) underestimate the re-operation rate.

We found a readmission rate of 4.4%; this aligns with a previous report from Turcotte et al., who reported readmission rates of 5.9% [26]. NORspine did not record readmission.

We did not find any statistical significant associations between postoperative complications and patient-reported clinical outcomes and satisfaction. We believe that most postoperative complications are time-limited and do not affect the clinical results in large heterogeneous populations. Our finding of no association between suffering from postoperative complications and satisfaction are not supported by the literature. Previous studies report lower patient satisfaction among surgical and medical patients who suffered from postoperative complications or medical adverse effects [4, 14, 21]. Patient satisfaction involves several aspects, including the quality of the treatment, clinical outcome, and more subtle aspects, such as the relationship between patients and healthcare personnel. Patients may still be content with the treatment if postoperative complications are recognised and treated.

The NORspine do not classify the severity of the complications. There are different classification systems, i.e.Clavien-Dindo classification grading) CDG), and Spine Adverse Effect System version 2 (SAVES-V2); however, a global standard have yet to be established for all spine registries [6, 18, 20]. Some spine registries record complications by patients, some by surgeons; hence, the heterogeneity of definitions and reporting makes comparisons between different registries problematic. A future goal could be to establish a standard system for grading and recording complications (or even a common international spine registry).

### Limitations

Our patients were not randomly selected, and the study population was sampled from four different hospitals with which the authors were affiliated and at which the authors had access to EPRs. Legal and practical reasons restricted this sampling. The study population and clinical results were, however, representative of the national LSS population (Tables 1 and 2) [1, 8, 9, 23, 31]. The loss to follow-up of 31% is also comparable to other register-based studies [7, 11, 13, 24], and we found no systematic differences between patients completing three months of follow-up and the entire study population. Loss to follow-up of 31% has not been shown to bias register outcomes systematically, and our follow-up rate was also sufficient to allow for a reliable analysis according to register recommendations by van Hoof [7, 11, 13, 24, 27].

We included only patients from the four treating centres also participating in the NORspine registry to make comparison between the data sources possible; this may have led to selection bias.

Large populations are needed to study complications as the prevalence of complications is low. This study included 474 patients, and a 3-month follow-up was available for 326 patients.

There is no gold standard for defining and registering postoperative complications after LSS surgery — the NORspine registry bases registration of complications on patient-reported questionnaires. Because few patients have medical education, patients are expected to classify postoperative complications differently than healthcare personnel (i.e., infections). Also, patients are not asked to report certain postoperative complications to NORspine, such as epidural hematoma and neurologic deficit or readmission. EPR was the only data source available for the complications above or events.

We have only reviewed patient records at the treating centres and did not have access to patient records from other treating centres or primary care institutions; hence, we may have missed some complications in the EPR review.

### Strenghts

We combined two different data sources and assessed both patient – and healthcare personnel-reported complications to gain a more reliable estimate of postoperative complications after LSS surgery. To our knowledge, this is the first study to combine different data sources to assess complications after spinal surgery. We did not formally assess the discrepancies between the two data sources since none of the two sources used may be defined as a gold standard.

## Conclusions

Reliable registration of complications after spine surgery is challenging; hence, it is essential to critically evaluate the method of registration when interpreting data on postoperative complications. Combining patient- and healthcare personnel-reported complications after LSS surgery revealed a higher rate of postoperative complications and reoperation rate than by each data source alone. Postoperative complications were not associated with patient-reported clinical outcome or satisfaction at three months after LSS surgery.

## Data Availability

Data can be provided.
